# Association between oxidative balance score and prostate specific antigen among older US adults

**DOI:** 10.3389/fpubh.2023.1336657

**Published:** 2024-01-22

**Authors:** Jintao Li, Chao Yang, Kui Xiang

**Affiliations:** The National Hospital of Enshi Tujia and Miao Autonomous Prefecture, Enshi, Hubei, China

**Keywords:** oxidative balance score, prostate specific antigen, older adults, NHANES, association

## Abstract

**Objective:**

Oxidative Balance Score (OBS) is an index affecting the oxidative stress of dietary and lifestyle factors. We aimed to explore the association of OBS with prostate specific antigen (PSA) among older males.

**Methods:**

A total of 5,136 samples were collected in this study to investigate the relationship between OBS and PSA from the National Health and Nutrition Examination Survey. Logistic regression models and restricted cubic spline were used to assess the associations between OBS and PSA.

**Results:**

Compared with the Q1 group, the odds ratios for the association between OBS and PSA were 1.005 (1.003, 1.009), 1.003 (1.001, 1.006), and 1.001 (0.978, 1.022) for Q2, Q3, and Q4, respectively. In the age-specific analyses, the association was significant among individuals aged 65 years old and over: the odds ratios for the association between OBS and PSA were 1.019 (1.005, 1.028), 1.028 (1.018, 1.039), and 1.038 (1.022, 1.049) for Q2, Q3, and Q4, respectively. But it was not significant among individuals aged less than 65 years old: the odds ratios for the association between OBS and PSA were 1.016 (0.995, 1.026), 1.015 (0.985, 1.022), and 0.988 (0.978, 1.016) for Q2, Q3, and Q4, respectively. The restricted cubic splines also indicated a nonlinear relationship between OBS and PSA among individuals aged 65 years old and over (*P*_overall_ = 0.006, *P*_nonlinear_ = 0.021).

**Conclusion:**

Our findings provide evidence that OBS is positively associated with higher levels of PSA among older adults. Further large-scale prospective cohort studies are needed to verify our findings.

## Introduction

Prostate cancer (PC) is the third most common cancer worldwide and one of the most common malignancies among men, alongside lung and colon tumors, resulting in an estimated 161,360 new cases of prostate cancer and 26,730 deaths in the United States in 2017 (1–3). Since the promotion of serum prostate-specific antigen (PSA) screening and the development of prostate biopsy in the late 1980s, the number of confirmed cases of prostate cancer increased significantly (4, 5). The incidence of prostate cancer reached a peak in 1992, decreased between 1992 and 1995, and then increased at a rate of about 1% per year (6). According to the latest studies, prostate cancer has become the second most common malignant tumor among male tumors, with an estimated 165, 000 newly diagnosed cases and 29,000 deaths in 2018 (7, 8).

Prostate-specific antigen (PSA) has become an indispensable tumor marker for the diagnosis of prostate cancer and has been widely used for disease monitoring, staging and diagnosis, and early diagnosis, in addition to being an immunohistochemical marker (9). Serum levels are also affected by factors such as age, prostate size, and ethnicity (10). The change of the prostate surrounding environment is also the main factor affecting the serum.

The occurrence and advancement of PC have been linked to oxidative stress, which is an imbalance between the antioxidant defense system’s generation and scavenging of reactive oxygen species (ROS) (11). Numerous dietary elements and lifestyle choices have been linked to oxidative stress. An individual’s oxidative/antioxidant state was evaluated using the Oxidative Balance Score (OBS), which was calculated by adding up the pro- and antioxidant components of dietary and lifestyle factors (12). OBS has been shown to be useful in treating a number of chronic conditions, such as hypertension and gastric reflux. Its ability to measure diet and lifestyle choices that may have a negative impact on health was particularly valuable in epidemiological research (13–15). It is unknown, however, if OBS and PSA are related.

Therefore, we carried out a cross-sectional investigation into the relationship between OBS and PSA in the current study. Based on data from National Health and Nutrition Examination Surveys, the study was carried out among adult US citizens.

## Methods

### Study population

Data for the study were gathered using questionnaires, interviews, physical examinations, and laboratory testing from the National Health and Nutrition Examination Surveys (NHANES 2003–2010) (16). An online description of NHANES can be found at https://wwwn.cdc.gov/nchs/nhanes/analyticguidelines.aspx. The National Center for Health Statistics Institutional Review Board approved the study, and written informed consent was obtained from each participant. First included in our study were 17,902 individuals with OBS data aged equal to or more than 20 years old from continuous NHANES (2003–2010) datasets. Women (*n* = 9,402), individuals without a PSA (*n* = 2,246), those without an OBS, and any missing basic data (*n* = 1,118) were also eliminated. Ultimately, our research encompassed 5,136 people in total. The study’s flowchart is shown in Supplementary Figure S1.

### Definition of OBS

Based on prior information about the relationship between nutrients or lifestyle factors and OS, 16 nutrients and four lifestyle factors were screened to calculate OBS, of which five were pro-oxidants and 15 were antioxidants (17, 18). Of these, 16 nutrients were calculated from the mean of the various ingredients from the nutrition interviews on day 1 and day 2 to calculate the quantile as the scoring threshold. Supplementary Table S1 shows the distribution scheme of OBS components. We divided the other components into three groups by sex-specific quantiles. The antioxidants were assigned to one-third of the group at points 0 to 2. The point distribution of the pro-oxidant was reversed, with 0 points representing the highest quantile and 2 points representing the lowest. Finally, the OBS scores of each participant were summed to get the final OBS value of each participant.

### Assessment of PSA

The most prevalent non-cutaneous cancer in males is prostate cancer, and total PSA testing has been identified as a tumor marker for cancer detection, diagnosis, and therapy. For men 40 years of age and above, the survey measures total prostate-specific antigen (PSA). Blood samples were subjected to PSA immunoassays utilizing the Hybritech test (Beckman Coulter, Fullerton, CA). A paramagnetic particle coated with a second mouse monoclonal anti-PSA antibody and a mouse monoclonal anti-PSA alkaline phosphatase conjugate were placed into a reaction vessel together with a second sample. While the monoclonal anti-PSA adjuvant interacts with several antigenic sites on the sample PSA, the PSA in the sample binds to the monoclonal anti-PSA immobilized on the solid phase. The amount of light produced is directly correlated with the PSA levels in the sample. The typical PSA level was 0–4 ng/mL (19).

### Covariates

Face-to-face interviews were used to gather information on age, gender, education levels, race, and ethnicity (Mexican American, Other Hispanic, Non-Hispanic White, Non-Hispanic Black, and others), family income-poverty ratios (PIR), smoking, and drinking. Physical examinations were used to determine body mass index (BMI). Weight (in kilos) divided by height (in meters squared) yielded the BMI. PIR was computed by dividing family (or individual) income by the survey year’s specific poverty guidelines, which differed according to the household size and the area. Lower PIR indicated a higher degree of poverty, whereas a ratio of one indicated the same income and level of poverty. Those who had smoked at least 100 cigarettes in their lifetime were considered smokers. Individuals who had ingested one or more alcoholic beverages throughout the preceding year were identified as current drinkers.

### Statistical analysis

For continuous variables, the mean (SD) was reported, and for categorical variables, the numbers (percentages) represented the basic features of the participants. For categorical or continuous variables, the *T* test or the Chi-square test was used. Four distinct scenarios were employed in logistic regression models to evaluate the relationships between OBS and PSA. Additionally, limited cubic splines were employed to evaluate the link between OBS and PSA’s dose and response. Age, BMI, sex, education, race/ethnicity, marital status, PIR, drinking and smoking habits, diabetes, hypertension, and marriage were among the covariates. Furthermore, age has been identified as a primary risk factor for PSA. Consequently, stratified analyses based on age—that is, age < 65 and age ≥ 65—were used to examine the relationship between OBS and PSA. Software from SAS Institute Inc., Cary, NC, version 9.4 was used for all statistical analyses. *p* < 0.05 was used as the threshold for significance in the two-sided statistical tests.

## Results

The basic characteristics of the subjects are presented in Table 1. A total of 5,136 participants were included in our study; the number of OBS in different quartiles was 1,290, 1,358, 1,214, and 1,194, respectively. And the mean PSA was 1.67, 1.84, 1.68, and 2.07, respectively. We found that all variables except for hypertension were statistically significant between different quartiles of OBS (*p* = 0.475). The characteristics of the subjects aged below 65 and equal to and over 65 are presented in Supplementary Tables S2, S3; the results were similar to the total population.

**Table 1:** The baseline characteristics of included samples by quartiles of the OBS.

All	Q1 (*N* = 1,290)	Q2 (*N* = 1,358)	Q3 (*N* = 1,214)	Q4 (*N* = 1,194)	*p*
**OBS value**	−0.48 (1.87)	5.50 (1.74)	11.4 (1.71)	18.3 (2.58)	
**Age**	61.6 (12.1)	60.6 (12.8)	59.1 (12.8)	57.1 (12.1)	<0.001
**Race**					<0.001
Mexican American	217 (16.8%)	249 (18.3%)	228 (18.8%)	163 (13.7%)	
Other Hispanic	102 (7.91%)	77 (5.67%)	78 (6.43%)	63 (5.28%)	
Non-Hispanic White	579 (44.9%)	742 (54.6%)	699 (57.6%)	809 (67.8%)	
Non-Hispanic Black	354 (27.4%)	250 (18.4%)	165 (13.6%)	131 (11.0%)	
Other Race	38 (2.95%)	40 (2.95%)	44 (3.62%)	28 (2.35%)	
**Education**					<0.001
Less than 9th grade	310 (24.0%)	219 (16.1%)	155 (12.8%)	90 (7.54%)	
9–11th grade	240 (18.6%)	211 (15.5%)	148 (12.2%)	109 (9.13%)	
High school graduate	338 (26.2%)	311 (22.9%)	290 (23.9%)	262 (21.9%)	
Some college or AA degree	268 (20.8%)	334 (24.6%)	325 (26.8%)	330 (27.6%)	
College graduate or above	133 (10.3%)	282 (20.8%)	296 (24.4%)	403 (33.8%)	
Refused	1 (0.08%)	0 (0.00%)	0 (0.00%)	0 (0.00%)	
Do not Know	0 (0.00%)	1 (0.07%)	0 (0.00%)	0 (0.00%)	
**PIR**	2.33 (1.52)	2.70 (1.58)	3.04 (1.60)	3.31 (1.61)	<0.001
**Marriage**					<0.001
Married	831 (64.4%)	929 (68.5%)	860 (70.8%)	870 (72.9%)	
Widowed	89 (6.90%)	98 (7.22%)	67 (5.52%)	52 (4.36%)	
Divorced	166 (12.9%)	131 (9.65%)	130 (10.7%)	120 (10.1%)	
Separated	48 (3.72%)	42 (3.10%)	31 (2.55%)	24 (2.01%)	
Never married	97 (7.52%)	83 (6.12%)	71 (5.85%)	68 (5.70%)	
Living with partner	58 (4.50%)	74 (5.45%)	53 (4.37%)	60 (5.03%)	
Refused	1 (0.08%)	0 (0.00%)	1 (0.08%)	0 (0.00%)	
Do not Know	0 (0.00%)	0 (0.00%)	1 (0.08%)	0 (0.00%)	
**Total PSA (ng/mL)**	1.67 (2.56)	1.84 (3.36)	1.68 (2.66)	2.07 (4.11)	0.006
**Smoking**					<0.001
Yes	728 (66.4%)	710 (63.2%)	611 (61.1%)	530 (54.5%)	
No	368 (33.5%)	414 (36.8%)	389 (38.9%)	443 (45.5%)	
Do not know	1 (0.09%)	0 (0.00%)	0 (0.00%)	0 (0.00%)	
**Alcohol**					<0.001
Yes	361 (31.4%)	354 (28.9%)	301 (27.7%)	259 (23.7%)	
No	790 (68.6%)	870 (71.0%)	787 (72.3%)	834 (76.3%)	
Do not know	0 (0.00%)	1 (0.08%)	0 (0.00%)	0 (0.00%)	
**Diabetes**					<0.001
Yes	272 (21.1%)	214 (15.8%)	176 (14.5%)	136 (11.4%)	
No	985 (76.4%)	1,103 (81.2%)	1,010 (83.2%)	1,035 (86.7%)	
Borderline	31 (2.40%)	39 (2.87%)	28 (2.31%)	23 (1.93%)	
Do not know	2 (0.16%)	2 (0.15%)	0 (0.00%)	0 (0.00%)	
**Hypertension**					0.475
Yes	58 (4.86%)	53 (4.31%)	52 (4.59%)	40 (3.58%)	
No	1,135 (95.1%)	1,176 (95.7%)	1,081 (95.4%)	1,077 (96.4%)	
**BMI (Kg/m** ^**2**^ **)**					<0.001
Normal (25<)	301 (23.3%)	395 (29.1%)	377 (31.1%)	386 (32.3%)	
Overweight (25 ≤ BMI < 30)	434 (33.6%)	496 (36.5%)	437 (36.0%)	464 (38.9%)	
Obesity (≥30)	555 (43.0%)	467 (34.4%)	400 (32.9%)	344 (28.8%)	
**OBS components**					
**Dietary OBS components**					
Calcium (mg/d)	0.20 (0.42)	0.60 (0.68)	1.04 (0.72)	1.56 (0.62)	<0.001
Magnesium (mg/d)	0.07 (0.26)	0.54 (0.59)	1.22 (0.56)	1.83 (0.39)	<0.001
Zinc (mg/d)	0.16 (0.40)	0.58 (0.67)	1.08 (0.69)	1.60 (0.58)	<0.001
Copper (mg/d)	0.11 (0.35)	0.57 (0.62)	1.13 (0.65)	1.72 (0.49)	<0.001
Selenium (mcg/d)	0.19 (0.42)	0.58 (0.65)	1.05 (0.75)	1.51 (0.67)	<0.001
Total fat (g/d)	0.33 (0.58)	0.66 (0.72)	0.98 (0.79)	1.33 (0.77)	<0.001
Dietary fiber (g/d)	0.24 (0.47)	0.75 (0.69)	1.27 (0.69)	1.73 (0.49)	<0.001
Carotene (RE/d)	0.62 (0.76)	0.98 (0.82)	1.16 (0.77)	1.47 (0.66)	<0.001
Riboflavin (mg/d)	0.19 (0.43)	0.61 (0.68)	1.09 (0.71)	1.67 (0.52)	<0.001
Niacin (mg/d)	0.13 (0.36)	0.49 (0.62)	1.00 (0.69)	1.62 (0.57)	<0.001
Vitamin B6 (mg/d)	0.09 (0.31)	0.56 (0.61)	1.13 (0.66)	1.75 (0.45)	<0.001
Total folate (mcg/d)	0.14 (0.38)	0.59 (0.63)	1.16 (0.67)	1.72 (0.49)	<0.001
Vitamin B12 (mcg/d)	0.26 (0.50)	0.70 (0.73)	1.06 (0.75)	1.56 (0.63)	<0.001
Vitamin C (mg/d)	0.46 (0.65)	0.89 (0.78)	1.12 (0.77)	1.53 (0.63)	<0.001
Vitamin E (ATE) (mg/d)	0.20 (0.45)	0.65 (0.70)	1.09 (0.72)	1.65 (0.54)	<0.001
Iron (mg/d)	0.20 (0.44)	0.61 (0.67)	1.13 (0.70)	1.62 (0.58)	<0.001
**Lifestyle OBS components**					
Leisure time physical activity	1.24 (0.42)	1.33 (0.47)	1.37 (0.48)	1.49 (0.50)	<0.001
Alcohol (g/d)	1.85 (0.46)	1.68 (0.63)	1.58 (0.70)	1.48 (0.77)	<0.001
Cotinine (ng/mL)	1.18 (0.79)	0.93 (0.81)	0.87 (0.81)	0.71 (0.78)	<0.001

OBS, oxidative balance score; BMI, body mass index; PIR, poverty-to-income ratio.

The association between the OBS and PSA is presented in Table 2. Compared with the quartile 1 group, the odds ratios for the association between OBS and PSA were 1.005 (1.003, 1.009), 1.003 (1.001, 1.006), and 1.001 (0.978, 1.022) for quartile 2, quartile 3, and quartile 4, respectively, after adjusting for other covariates. In the age-specific analyses, the association was significant among individuals 65 years old and over, not among individuals aged less than 65 years old. For individuals aged 65 years old and over, the odds ratios for the association between OBS and PSA were 1.019 (1.005, 1.028), 1.028 (1.018, 1.039), and 1.038 (1.022, 1.049) for Q2, Q3, and Q4, respectively, after adjusting for other covariates. For individuals aged less than65 years old, the odds ratios for the association between OBS and PSA were 1.016 (0.995, 1.026), 1.015 (0.985, 1.022), and 0.988 (0.978, 1.016) for Q2, Q3, and Q4, respectively.

**Table 2:** Odds ratio for the association between the OBS and PSA.

	Q1	Q2	Q3	Q4
All				
Model 1	ref	1.016 (1.015, 1.016)	1.008 (1.008, 1.009)	0.999 (0.999, 1.000)
Model 2	ref	1.013 (1.001, 1.025)	1.007 (1.000, 1.012)	1.002 (0.998, 1.022)
Model 3	ref	1.007 (1.002, 1.011)	1.003 (1.001, 1.008)	1.002 (0.988, 1.022)
Model 4	ref	1.005 (1.003, 1.009)	1.003 (1.001, 1.006)	1.001 (0.978, 1.022)
<65				
Model 1	ref	1.012 (1.012, 1.013)	0.998 (0.998, 1.098)	0.985 (0.987, 1.099)
Model 2	ref	1.008 (0.985, 1.013)	1.003 (0.998, 1.006)	0.993 (0.978, 1.002)
Model 3	ref	1.016 (0.988, 1.012)	1.002 (0.978, 1.026)	0.985 (0.966, 1.085)
Model 4	ref	1.016 (0.995, 1.026)	1.015 (0.985, 1.022)	0.988 (0.978, 1.016)
≥65				
Model 1	ref	1.011 (1.010, 1.011)	1.021 (1.016, 1.032)	1.032 (1.018, 1.041)
Model 2	ref	1.001 (1.000, 1.002)	1.012 (1.002, 1.026)	1.026 (1.008, 1.036)
Model 3	ref	1.018 (1.005, 1.021)	1.025 (1.014, 1.032)	1.035 (1.019, 1.048)
Model 4	ref	1.019 (1.005, 1.028)	1.028 (1.018, 1.039)	1.038 (1.022, 1.049)

Model 1: unadjusted.

Model 2: adjusted for sex, age (years, continuous), education (less than high school, high school graduate, some college and above), race (non-Hispanic White, non-Hispanic Black, Mexican American, other), Marriage (married, widowed, divorced, separated, never married, living with partner), and PIR.

Model 3: Model 2 plus self-reported alcohol status (yes, no) and self-reported smoking status (yes, no).

Model 4: Model 3 plus adjusted for BMI, self-reported hypertension (yes, no), and self-reported diabetes (yes, no).

OBS, oxidative balance score; PSA, prostate specific antigen.

**Figure 1:**
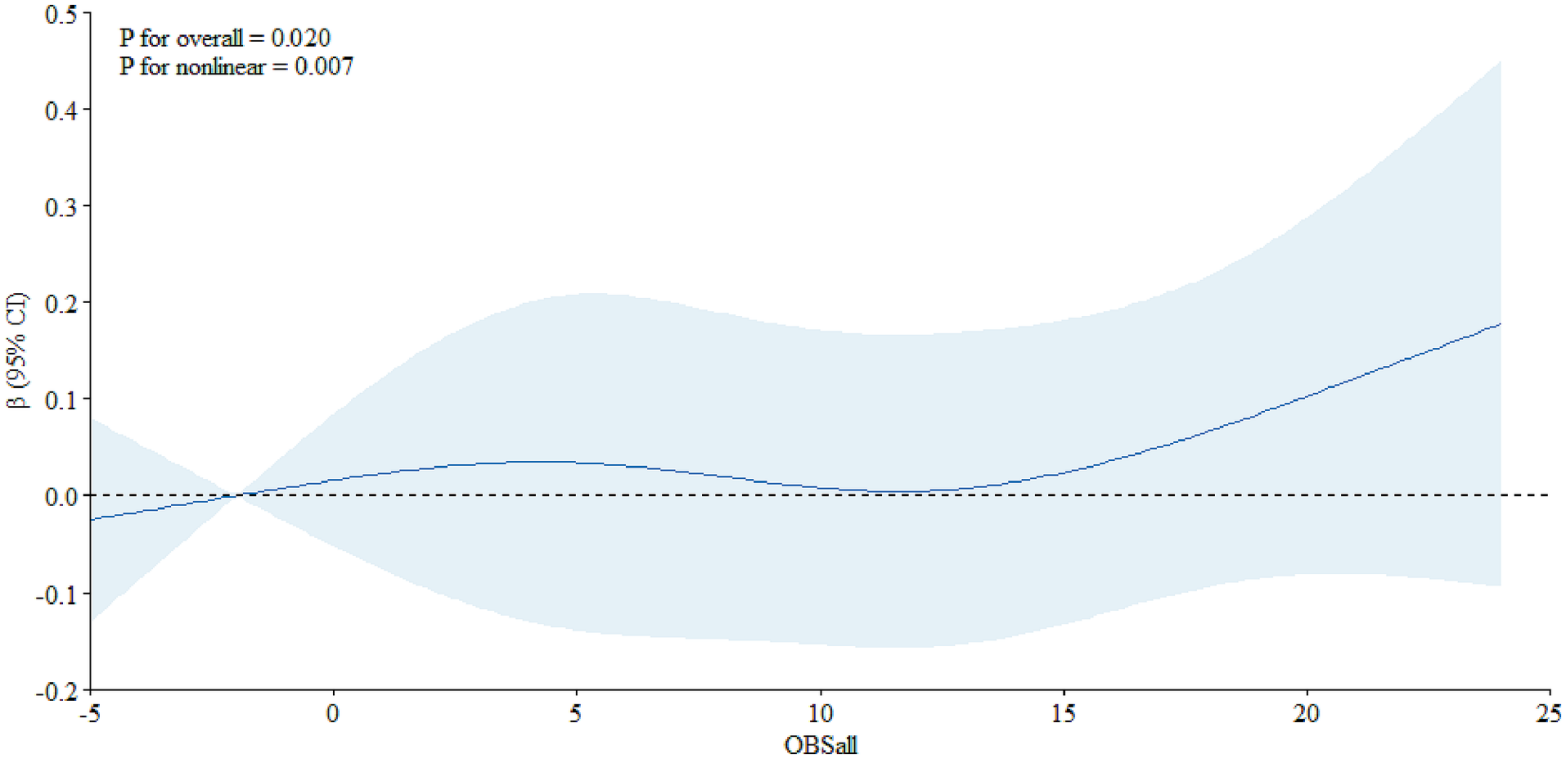
The dose–response relationship between the OBS and PSA among all the participants.

**Figure 2:**
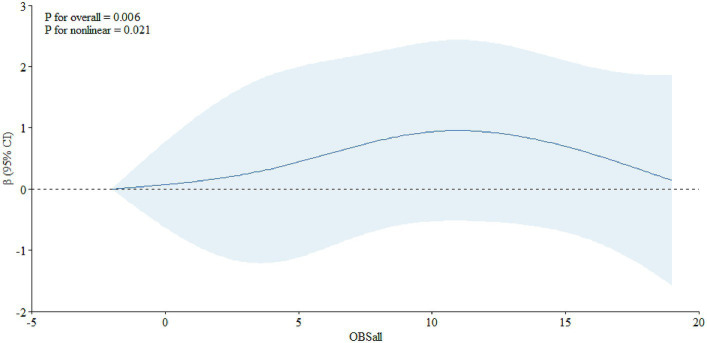
The dose–response relationship between the OBS and PSA among individuals aged 65 years and over.

**Figure 3:**
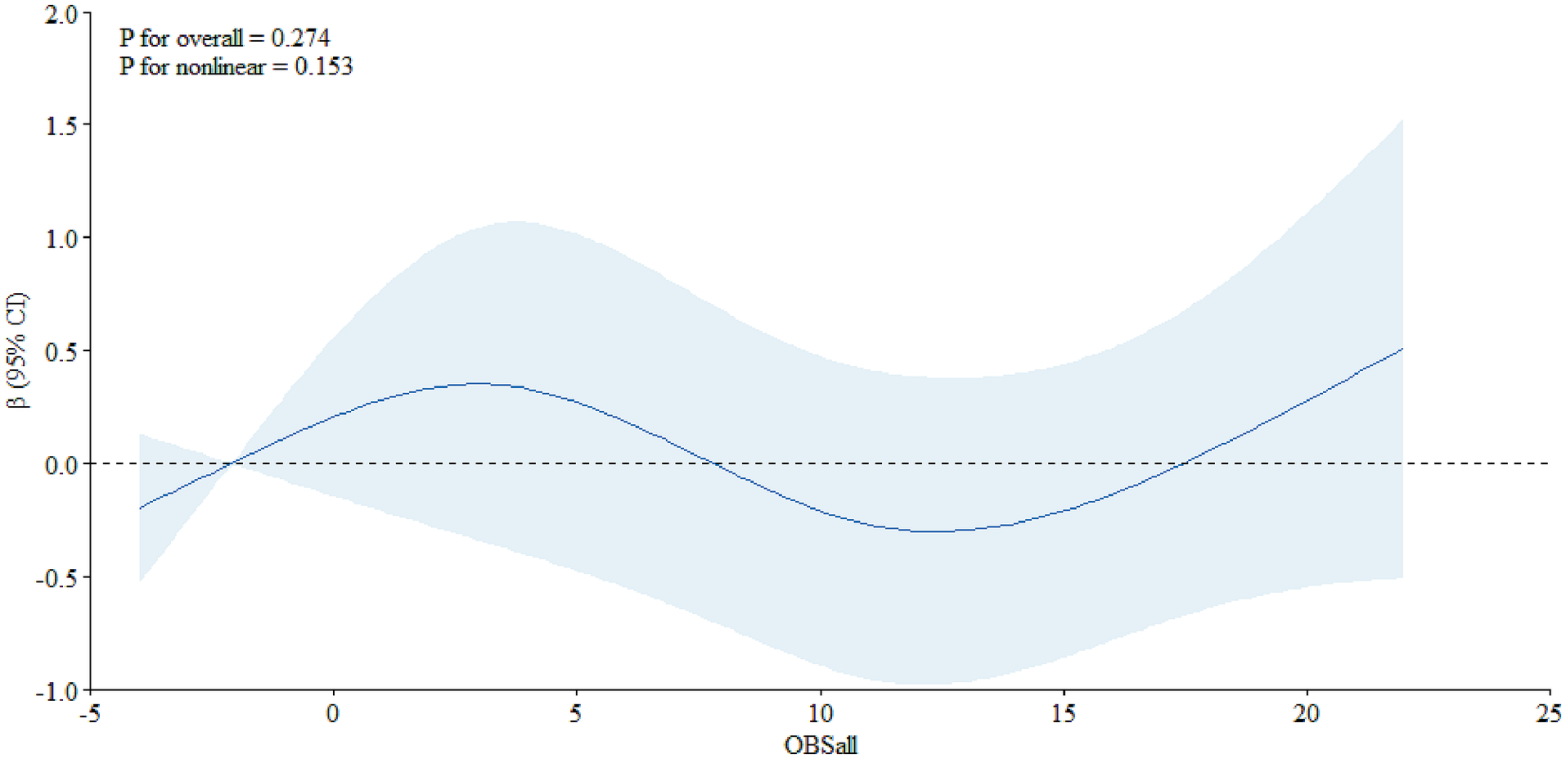
The dose–response relationship between the OBS and PSA among individuals aged less than 65.

In addition, we also assessed the dose–response relationship between OBS and PSA. We found a non-linear relationship between OBS and PSA in the total population (Figure 1, *P*_overall_ = 0.020, *P*_nonlinear_ = 0.007) and, in the age-specific analyses, it was significant among individuals aged 65 years and older (Figure 2, *P*_overall_ = 0.006, *P*_nonlinear_ = 0.021), not among individuals aged below 65 years old (Figure 3, *P*_overall_ = 0.274, *P*_nonlinear_ = 0.153).

## Discussion

In the present study, we found that OBS was positively associated with PSA among older adults, after adjusting for other covariates. Meanwhile, we also explored the dose–response relationship between them and found a non-linear relationship between OBS and PSA among older adults.

The results of the study have important implications for both public health and clinical settings, as PC is not only a public health issue but also an important clinical disease. It indicated that physicians should pay attention to reducing oxidative stress, which may be helpful to reduce the level of PSA, and then reduce the occurrence of PC. And from a public health perspective, adequate antioxidant food intake may be helpful for the prevention of PC for men, especially for older adults.

In the subgroup analysis, we found that the association was significant among individuals aged 65 years old and older, but it was not significant among those aged below 65 years old. We believe several factors may contribute to this phenomenon. First, studies have shown that the level of oxidative stress would increase with the increase of age, as the process of aging in the human body itself is accompanied by oxidation of the body (20, 21). Secondly, when the human body is aging, the organ function of the body gradually declines and the absorption capacity of antioxidant is weakened, which could result in the high level of oxidative stress (22, 23).

The mechanism underlying the positive association between OBS and PSA is unclear. The process of oxidative stress is usually induced by the elevation of cellular ROS, and immune function, oxidative metabolism, and mitochondrial bioenergetics could continuously generate ROS (24, 25). It is usually in hypochlorous acid, hydroxyl radical, superoxide anion, and other substances related with cell growth, differentiation, and death (26). Previous studies have reported that several complex cell signaling pathways are involved in the progression and incidence of PC with animal models and cell culture experiments (27). Actually, several factors could cause oxidative free radicals, including delaying the recruitment of p53 and regulating androgens and inflammation (28). And studies have indicated that the increase of ROS accumulation and production in prostate cancer cells was related with serum androgens (29).

There are a few more restrictions that must be mentioned. Firstly, as the study is only cross-sectional, a causal relationship between OBS and PSA cannot be established. Second, we were unable to completely rule out the possibility of unassessed confounders even after adjusting for a variety of plausible risk factors in our investigation. Thirdly, as this study only included adult US citizens, care should be taken when extrapolating the results to other demographics.

## Conclusion

In the present study, we found that OBS is positively associated with PSA among older adults, and a nonlinear relationship could also be found between OBS and PSA. Further large-scale prospective cohort studies are needed to verify our findings and explore the association between OBS and PSA.

## Data availability statement

The original contributions presented in the study are included in the article/Supplementary material, further inquiries can be directed to the corresponding author.

## Author contributions

JL: Formal analysis, Methodology, Writing – original draft. CY: Methodology, Validation, Visualization, Writing – original draft. KX: Conceptualization, Software, Supervision, Writing – review & editing.
