# Supplementary material for: Association between oxidative balance score and prostate specific antigen among older US adults
Source: Front Public Health. 2024 Jan 22;11:1336657. doi: 10.3389/fpubh.2023.1336657 (PMC10838971; doi:10.3389/fpubh.2023.1336657)

**Table S1 Oxidative balance score assignment scheme**

| **OBS components** | **Property** | | **Male** | | | | **Female** | | |
| --- | --- | --- | --- | --- | --- | --- | --- | --- | --- |
| **Dietary OBS components** | | 0 | | 1 | | 2 | 0 | 1 | 2 |
| Calcium (mg/d) | A | | <751 | | 751-1167 | ≥1167 | <617.5 | 617.5-938.5 | ≥938.5 |
| Magnesium (mg/d) | A | | <265.5 | | 265.5-365.5 | ≥365.5 | <203.5 | 203.5-286 | ≥286 |
| Zinc (mg/d) | A | | <10.69 | | 10.69-15.78 | ≥15.78 | <7.62 | 7.62-11.08 | ≥11.08 |
| Copper (mg/d) | A | | <1.142 | | 1.14-1.61 | ≥1.61 | <0.89 | 0.89-1.26 | ≥1.26 |
| Selenium (mcg/d) | A | | <104 | | 104-146.4 | ≥146.4 | <73.75 | 73.75-103.15 | ≥103.15 |
| Dietary fiber (g/d) | A | | <13.25 | | 13.25-20.3 | ≥20.3 | <10.95 | 10.95-16.45 | ≥16.45 |
| Carotene (RE/d) | A | | <154.75 | | 154.75-628.42 | ≥628.42 | <169.92 | 169.92-713.25 | ≥713.25 |
| Riboflavin (mg/d) | A | | <1.954 | | 1.95-2.81 | ≥2.81 | <1.49 | 1.49-2.12 | ≥2.12 |
| Niacin (mg/d) | A | | <23.58 | | 23.58-33.31 | ≥33.31 | <16.44 | 16.44-23.02 | ≥23.02 |
| Vitamin B6 (mg/d) | A | | <1.77 | | 1.768-2.591 | ≥2.59 | <1.28 | 1.28-1.88 | ≥1.88 |
| Total folate (mcg/d) | A | | <346.5 | | 346.5-516 | ≥516 | <269.5 | 269.5-392.5 | ≥392.5 |
| Vitamin B12 (mcg/d) | A | | <4.04 | | 4.04-6.82 | ≥6.82 | <2.83 | 2.83-4.86 | ≥4.86 |
| Vitamin C (mg/d) | A | | <44.7 | | 44.7-105.4 | ≥105.4 | <42.2 | 42.2-93.35 | ≥93.35 |
| Vitamin E (ATE) (mg/d) | A | | <5.875 | | 5.88-9.19 | ≥9.19 | <4.64 | 4.64-7.41 | ≥7.41 |
| Total fat (g/d) | P | | <72.17 | | 72.17-107.03 | ≥107.03 | <51.73 | 51.73-76.03 | ≥76.03 |
| Iron (mg/d) | P | | <13.75 | | 13.75-20.07 | ≥20.07 | <10.33 | 10.33-14.85 | ≥14.85 |
| **Lifestyle OBS components** | | | | | | | | | |
| Leisure time  physical activity | A | | <0 | | 0-180 | ≥180 | <0 | 0-180 | ≥180 |
| Alcohol (g/d) | P | | <0 | | 0-30 | ≥30 | <0 | 0-15 | ≥15 |
| BMI | P | | <25.84 | | 25.84-30.02 | ≥30.02 | <25.39 | 25.39-31.27 | ≥31.27 |
| Cotinine (ng/mL) | P | | <0.03 | | 0.03-2.32 | ≥2.32 | <0.02 | 0.02-0.14 | ≥0.14 |

Abbreviations: OBS, oxidative balance score; BMI, body mass index.

**Table S2 The baseline characteristics of participants under 65 years old by quartiles of the OBS**

| **Age<65** | **Q1 (N=749)** | **Q2 (N=814)** | **Q3 (N=793)** | **Q4 (N=850)** | ***P*** |
| --- | --- | --- | --- | --- | --- |
| **OBS value** | -0.54 (1.90) | 5.58 (1.74) | 11.4 (1.69) | 18.3 (2.55) |  |
| **Age** | 53.0 (7.11) | 51.7 (7.30) | 51.2 (7.28) | 50.7 (6.96) | <0.001 |
| **Race** |  |  |  |  | <0.001 |
| Mexican American | 133 (17.8%) | 182 (22.4%) | 177 (22.3%) | 134 (15.8%) |  |
| Other Hispanic | 69 (9.21%) | 51 (6.27%) | 58 (7.31%) | 55 (6.47%) |  |
| Non-Hispanic White | 291 (38.9%) | 372 (45.7%) | 389 (49.1%) | 534 (62.8%) |  |
| Non-Hispanic Black | 229 (30.6%) | 184 (22.6%) | 132 (16.6%) | 105 (12.4%) |  |
| Other Race | 27 (3.60%) | 25 (3.07%) | 37 (4.67%) | 22 (2.59%) |  |
| **Education** |  |  |  |  | <0.001 |
| Less than 9th grade | 129 (17.2%) | 117 (14.4%) | 94 (11.9%) | 52 (6.12%) |  |
| 9-11th grade | 142 (19.0%) | 126 (15.5%) | 95 (12.0%) | 77 (9.06%) |  |
| High school graduate | 210 (28.0%) | 192 (23.6%) | 184 (23.2%) | 180 (21.2%) |  |
| Some college or AA degree | 182 (24.3%) | 215 (26.4%) | 231 (29.1%) | 252 (29.6%) |  |
| College graduate or above | 85 (11.3%) | 164 (20.1%) | 189 (23.8%) | 289 (34.0%) |  |
| Refused | 1 (0.13%) | 0 (0.00%) | 0 (0.00%) | 0 (0.00%) |  |
| **PIR** | 2.48 (1.61) | 2.78 (1.66) | 3.08 (1.64) | 3.37 (1.66) | <0.001 |
| **Marriage** |  |  |  |  | <0.001 |
| Married | 468 (62.5%) | 536 (65.9%) | 547 (69.0%) | 619 (72.8%) |  |
| Widowed | 12 (1.60%) | 15 (1.85%) | 13 (1.64%) | 10 (1.18%) |  |
| Divorced | 114 (15.2%) | 91 (11.2%) | 96 (12.1%) | 88 (10.4%) |  |
| Separated | 34 (4.54%) | 36 (4.43%) | 26 (3.28%) | 19 (2.24%) |  |
| Never married | 74 (9.88%) | 71 (8.73%) | 61 (7.69%) | 60 (7.06%) |  |
| Living with partner | 46 (6.14%) | 64 (7.87%) | 48 (6.05%) | 54 (6.35%) |  |
| Refused | 1 (0.13%) | 0 (0.00%) | 1 (0.13%) | 0 (0.00%) |  |
| Don't Know | 0 (0.00%) | 0 (0.00%) | 1 (0.13%) | 0 (0.00%) |  |
| **Total PSA (ng/mL)** | 1.28 (1.71) | 1.17 (1.34) | 1.22 (1.63) | 1.25 (1.51) | 0.578 |
| **Smoking** |  |  |  |  | <0.001 |
| Yes | 396 (62.8%) | 401 (59.3%) | 391 (59.2%) | 348 (49.8%) |  |
| No | 235 (37.2%) | 275 (40.7%) | 270 (40.8%) | 351 (50.2%) |  |
| **Alcohol** |  |  |  |  | 0.006 |
| Yes | 223 (32.8%) | 230 (31.4%) | 221 (30.4%) | 195 (25.0%) |  |
| No | 457 (67.2%) | 503 (68.6%) | 506 (69.6%) | 584 (75.0%) |  |
| **Diabetes** <0.001 | | | | | |
| Yes | 132 (17.6%) | 112 (13.8%) | 87 (11.0%) | 68 (8.00%) |  |
| No | 598 (79.8%) | 678 (83.3%) | 690 (87.0%) | 763 (89.8%) |  |
| Borderline | 19 (2.54%) | 23 (2.83%) | 16 (2.02%) | 19 (2.24%) |  |
| Don't know | 0 (0.00%) | 1 (0.12%) | 0 (0.00%) | 0 (0.00%) |  |
| **Hypertension** |  |  |  |  | 0.42 |
| Yes | 40 (5.68%) | 41 (5.51%) | 37 (4.96%) | 32 (3.99%) |  |
| No | 664 (94.3%) | 703 (94.5%) | 709 (95.0%) | 770 (96.0%) |  |
| **BMI (Kg/m^2^)** |  |  |  |  | <0.001 |
| Normal(25<) | 154 (20.6%) | 220 (27.0%) | 223 (28.1%) | 266 (31.3%) |  |
| Overweight(25≤BMI<30) | 246 (32.8%) | 300 (36.9%) | 284 (35.8%) | 325 (38.2%) |  |
| Obesity(≥30) | 349 (46.6%) | 294 (36.1%) | 286 (36.1%) | 259 (30.5%) |  |
| **OBS components** | |  |  |  |  |
| **Dietary OBS components** | |  |  |  |  |
| Calcium (mg/d) | 0.23 (0.45) | 0.61 (0.69) | 1.07 (0.72) | 1.60 (0.59) | <0.001 |
| Magnesium (mg/d) | 0.09 (0.30) | 0.62 (0.60) | 1.28 (0.55) | 1.87 (0.34) | <0.001 |
| Zinc (mg/d) | 0.19 (0.43) | 0.68 (0.70) | 1.13 (0.68) | 1.64 (0.56) | <0.001 |
| Copper (mg/d) | 0.14 (0.38) | 0.62 (0.63) | 1.18 (0.64) | 1.77 (0.45) | <0.001 |
| Selenium (mcg/d) | 0.25 (0.47) | 0.71 (0.67) | 1.18 (0.72) | 1.63 (0.59) | <0.001 |
| Total fat (g/d) | 0.40 (0.62) | 0.77 (0.74) | 1.13 (0.79) | 1.46 (0.72) | <0.001 |
| Dietary fiber (g/d) | 0.23 (0.48) | 0.75 (0.71) | 1.23 (0.71) | 1.72 (0.50) | <0.001 |
| Carotene (RE/d) | 0.56 (0.73) | 0.89 (0.81) | 1.07 (0.78) | 1.43 (0.68) | <0.001 |
| Riboflavin (mg/d) | 0.20 (0.45) | 0.62 (0.69) | 1.08 (0.73) | 1.66 (0.52) | <0.001 |
| Niacin (mg/d) | 0.16 (0.40) | 0.61 (0.65) | 1.13 (0.68) | 1.66 (0.55) | <0.001 |
| Vitamin B6 (mg/d) | 0.10 (0.32) | 0.58 (0.62) | 1.16 (0.66) | 1.74 (0.46) | <0.001 |
| Total folate (mcg/d) | 0.15 (0.38) | 0.61 (0.61) | 1.17 (0.68) | 1.71 (0.49) | <0.001 |
| Vitamin B12 (mcg/d) | 0.27 (0.50) | 0.69 (0.73) | 1.05 (0.76) | 1.56 (0.64) | <0.001 |
| Vitamin C (mg/d) | 0.41 (0.65) | 0.82 (0.79) | 1.07 (0.79) | 1.50 (0.65) | <0.001 |
| Vitamin E (ATE) (mg/d) | 0.23 (0.49) | 0.66 (0.71) | 1.11 (0.72) | 1.67 (0.53) | <0.001 |
| Iron (mg/d) | 0.21 (0.44) | 0.63 (0.66) | 1.14 (0.69) | 1.62 (0.58) | <0.001 |
| **Lifestyle OBS components** | |  |  |  |  |
| Leisure time physical activity | 1.26 (0.44) | 1.33 (0.47) | 1.37 (0.48) | 1.50 (0.50) | <0.001 |
| Alcohol (g/d) | 1.83 (0.49) | 1.67 (0.65) | 1.55 (0.72) | 1.46 (0.79) | <0.001 |
| Cotinine (ng/mL) | 1.31 (0.76) | 1.06 (0.81) | 1.00 (0.83) | 0.79 (0.80) | <0.001 |

Abbreviations: OBS, oxidative balance score; BMI, body mass index; PIR, poverty-to-income ratio.

**Table S3 The baseline characteristics of participants equal to and over 65 years old by quartiles of the OBS**

| **Age>=65** | **Q1 (N=541)** | **Q2 (N=544)** | **Q3 (N=421)** | **Q4 (N=344)** | ***P*** |
| --- | --- | --- | --- | --- | --- |
| **OBS value** | -0.39 (1.84) | 5.38 (1.74) | 11.5 (1.74) | 18.2 (2.64) |  |
| **Age** | 73.6 (5.73) | 73.8 (5.93) | 74.0 (5.84) | 73.0 (5.88) | 0.075 |
| **Race** |  |  |  |  | <0.001 |
| Mexican American | 84 (15.5%) | 67 (12.3%) | 51 (12.1%) | 29 (8.43%) |  |
| Other Hispanic | 33 (6.10%) | 26 (4.78%) | 20 (4.75%) | 8 (2.33%) |  |
| Non-Hispanic White | 288 (53.2%) | 370 (68.0%) | 310 (73.6%) | 275 (79.9%) |  |
| Non-Hispanic Black | 125 (23.1%) | 66 (12.1%) | 33 (7.84%) | 26 (7.56%) |  |
| Other Race | 11 (2.03%) | 15 (2.76%) | 7 (1.66%) | 6 (1.74%) |  |
| **Education** |  |  |  |  | <0.001 |
| Less than 9th grade | 181 (33.5%) | 102 (18.8%) | 61 (14.5%) | 38 (11.0%) |  |
| 9-11th grade | 98 (18.1%) | 85 (15.6%) | 53 (12.6%) | 32 (9.30%) |  |
| High school graduate | 128 (23.7%) | 119 (21.9%) | 106 (25.2%) | 82 (23.8%) |  |
| Some college or AA degree | 86 (15.9%) | 119 (21.9%) | 94 (22.3%) | 78 (22.7%) |  |
| College graduate or above | 48 (8.87%) | 118 (21.7%) | 107 (25.4%) | 114 (33.1%) |  |
| Don't Know | 0 (0.00%) | 1 (0.18%) | 0 (0.00%) | 0 (0.00%) |  |
| **PIR** | 2.12 (1.35) | 2.58 (1.44) | 2.96 (1.52) | 3.16 (1.46) | <0.001 |
| **Marriage** |  |  |  |  | 0.318 |
| Married | 363 (67.1%) | 393 (72.2%) | 313 (74.3%) | 251 (73.0%) |  |
| Widowed | 77 (14.2%) | 83 (15.3%) | 54 (12.8%) | 42 (12.2%) |  |
| Divorced | 52 (9.61%) | 40 (7.35%) | 34 (8.08%) | 32 (9.30%) |  |
| Separated | 14 (2.59%) | 6 (1.10%) | 5 (1.19%) | 5 (1.45%) |  |
| Never married | 23 (4.25%) | 12 (2.21%) | 10 (2.38%) | 8 (2.33%) |  |
| Living with partner | 12 (2.22%) | 10 (1.84%) | 5 (1.19%) | 6 (1.74%) |  |
| **Total PSA(ng/mL)** | 2.53 (3.77) | 2.73 (3.94) | 2.84 (4.88) | 3.16 (5.85) | 0.029 |
| **Smoking** |  |  |  |  | 0.256 |
| Yes | 332 (71.2%) | 309 (69.0%) | 220 (64.9%) | 182 (66.4%) |  |
| No | 133 (28.5%) | 139 (31.0%) | 119 (35.1%) | 92 (33.6%) |  |
| Don't know | 1 (0.21%) | 0 (0.00%) | 0 (0.00%) | 0 (0.00%) |  |
| **Alcohol** |  |  |  |  | 0.025 |
| Yes | 138 (29.3%) | 124 (25.2%) | 80 (22.2%) | 64 (20.4%) |  |
| No | 333 (70.7%) | 367 (74.6%) | 281 (77.8%) | 250 (79.6%) |  |
| Don't know | 0 (0.00%) | 1 (0.20%) | 0 (0.00%) | 0 (0.00%) |  |
| **Diabetes** <0.001 | | | | | |
| Yes | 140 (25.9%) | 102 (18.8%) | 89 (21.1%) | 68 (19.8%) |  |
| No | 387 (71.5%) | 425 (78.1%) | 320 (76.0%) | 272 (79.1%) |  |
| Borderline | 12 (2.22%) | 16 (2.94%) | 12 (2.85%) | 4 (1.16%) |  |
| Don't know | 2 (0.37%) | 1 (0.18%) | 0 (0.00%) | 0 (0.00%) |  |
| **Hypertension** |  |  |  |  | 0.528 |
| Yes | 18 (3.68%) | 12 (2.47%) | 15 (3.88%) | 8 (2.54%) |  |
| No | 471 (96.3%) | 473 (97.5%) | 372 (96.1%) | 307 (97.5%) |  |
| **BMI(Kg/m2)** |  |  |  |  | <0.001 |
| Normal(25<) | 147 (27.2%) | 175 (32.2%) | 154 (36.6%) | 120 (34.9%) |  |
| Overweight(25≤BMI<30) | 188 (34.8%) | 196 (36.0%) | 153 (36.3%) | 139 (40.4%) |  |
| Obesity(≥30) | 206 (38.1%) | 173 (31.8%) | 114 (27.1%) | 85 (24.7%) |  |
| **OBS components** | |  |  |  |  |
| **Dietary OBS components** | |  |  |  |  |
| Calcium (mg/d) | 0.16 (0.38) | 0.60 (0.67) | 1.00 (0.71) | 1.44 (0.66) | <0.001 |
| Magnesium (mg/d) | 0.04 (0.19) | 0.42 (0.55) | 1.10 (0.55) | 1.72 (0.48) | <0.001 |
| Zinc (mg/d) | 0.11 (0.33) | 0.42 (0.61) | 0.98 (0.71) | 1.52 (0.60) | <0.001 |
| Copper (mg/d) | 0.07 (0.28) | 0.49 (0.61) | 1.04 (0.67) | 1.60 (0.57) | <0.001 |
| Selenium (mcg/d) | 0.10 (0.32) | 0.40 (0.59) | 0.82 (0.73) | 1.20 (0.76) | <0.001 |
| Total fat (g/d) | 0.24 (0.49) | 0.49 (0.64) | 0.70 (0.72) | 1.03 (0.80) | <0.001 |
| Dietary fiber (g/d) | 0.24 (0.46) | 0.75 (0.65) | 1.34 (0.66) | 1.75 (0.46) | <0.001 |
| Carotene (RE/d) | 0.70 (0.79) | 1.13 (0.82) | 1.34 (0.71) | 1.56 (0.61) | <0.001 |
| Riboflavin (mg/d) | 0.17 (0.41) | 0.61 (0.67) | 1.11 (0.67) | 1.67 (0.52) | <0.001 |
| Niacin (mg/d) | 0.07 (0.28) | 0.31 (0.51) | 0.76 (0.65) | 1.52 (0.62) | <0.001 |
| Vitamin B6 (mg/d) | 0.08 (0.29) | 0.52 (0.59) | 1.09 (0.65) | 1.76 (0.45) | <0.001 |
| Total folate (mcg/d) | 0.13 (0.38) | 0.57 (0.64) | 1.13 (0.66) | 1.74 (0.48) | <0.001 |
| Vitamin B12 (mcg/d) | 0.25 (0.50) | 0.71 (0.72) | 1.07 (0.73) | 1.58 (0.62) | <0.001 |
| Vitamin C (mg/d) | 0.52 (0.66) | 1.00 (0.74) | 1.23 (0.72) | 1.59 (0.55) | <0.001 |
| Vitamin E (ATE) (mg/d) | 0.16 (0.39) | 0.63 (0.70) | 1.07 (0.72) | 1.62 (0.57) | <0.001 |
| Iron (mg/d) | 0.18 (0.43) | 0.57 (0.69) | 1.10 (0.71) | 1.63 (0.58) | <0.001 |
| **Lifestyle OBS components** | |  |  |  |  |
| Leisure time physical activity | 1.21 (0.41) | 1.31 (0.46) | 1.37 (0.48) | 1.48 (0.50) | <0.001 |
| Alcohol (g/d) | 1.87 (0.41) | 1.71 (0.60) | 1.63 (0.64) | 1.53 (0.73) | <0.001 |
| Cotinine (ng/mL) | 1.00 (0.79) | 0.72 (0.76) | 0.63 (0.73) | 0.49 (0.67) | <0.001 |

Abbreviations: OBS, oxidative balance score; BMI, body mass index; PIR, poverty-to-income ratio.

**Figure S1 Flowchart of the study**


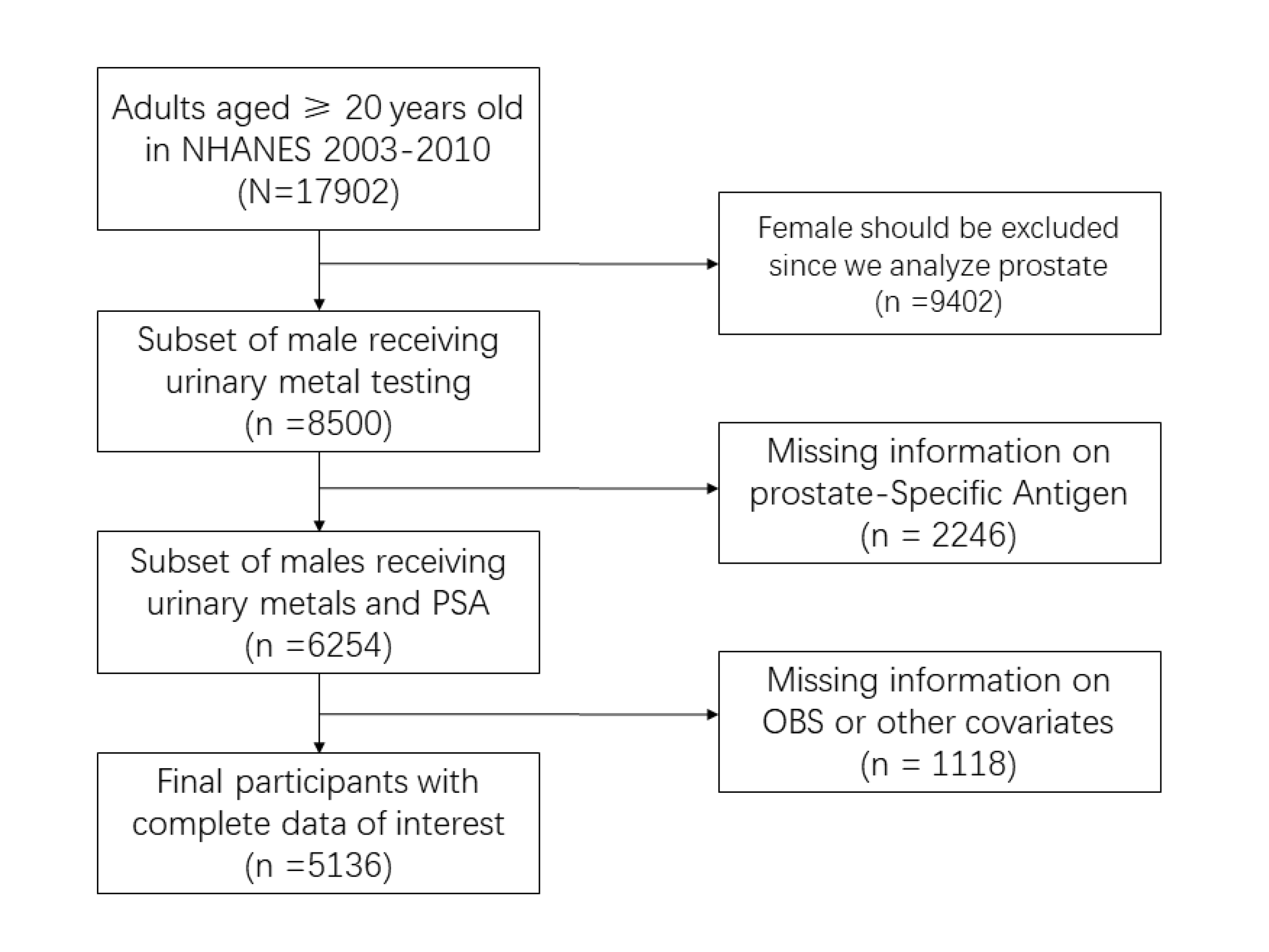

Supplement: Supplementary file 1 [file Data_Sheet_1.docx]
